# Far-UVC light (222 nm) efficiently and safely inactivates airborne human coronaviruses

**DOI:** 10.1038/s41598-020-67211-2

**Published:** 2020-06-24

**Authors:** Manuela Buonanno, David Welch, Igor Shuryak, David J. Brenner

**Affiliations:** Center for Radiological Research, Columbia University Irving Medical Center, New York, New York 10032 USA

**Keywords:** SARS-CoV-2, Microbiology, Applied microbiology

## Abstract

A direct approach to limit airborne viral transmissions is to inactivate them within a short time of their production. Germicidal ultraviolet light, typically at 254 nm, is effective in this context but, used directly, can be a health hazard to skin and eyes. By contrast, far-UVC light (207–222 nm) efficiently kills pathogens potentially without harm to exposed human tissues. We previously demonstrated that 222-nm far-UVC light efficiently kills airborne influenza virus and we extend those studies to explore far-UVC efficacy against airborne human coronaviruses alpha HCoV-229E and beta HCoV-OC43. Low doses of 1.7 and 1.2 mJ/cm^2^ inactivated 99.9% of aerosolized coronavirus 229E and OC43, respectively. As all human coronaviruses have similar genomic sizes, far-UVC light would be expected to show similar inactivation efficiency against other human coronaviruses including SARS-CoV-2. Based on the beta-HCoV-OC43 results, continuous far-UVC exposure in occupied public locations at the current regulatory exposure limit (~3 mJ/cm^2^/hour) would result in ~90% viral inactivation in ~8 minutes, 95% in ~11 minutes, 99% in ~16 minutes and 99.9% inactivation in ~25 minutes. Thus while staying within current regulatory dose limits, low-dose-rate far-UVC exposure can potentially safely provide a major reduction in the ambient level of airborne coronaviruses in occupied public locations.

## Introduction

Coronavirus disease 2019 (COVID-19) was first reported in December 2019 and then characterized as a pandemic by the World Health Organization on March 11, 2020. Despite extensive efforts to contain the spread of the disease, it has spread worldwide with over 5.3 million confirmed cases and over 340,000 confirmed deaths as of May 25, 2020^1^. Transmission of SARS-CoV-2, the beta coronavirus causing COVID-19, is believed to be both through direct contact and airborne routes, and studies of SARS-CoV-2 stability have shown viability in aerosols for at least 3 hours^2^. Given the rapid spread of the disease, including through asymptomatic carriers^3^, it is of clear importance to explore practical mitigation technologies that can inactivate the airborne virus in public locations and thus limit airborne transmission.

Ultraviolet (UV) light exposure is a direct antimicrobial approach^4^ and its effectiveness against different strains of airborne viruses has long been established^5^. The most commonly employed type of UV light for germicidal applications is a low pressure mercury-vapor arc lamp, emitting around 254 nm; more recently xenon lamp technology has been used, which emits broad UV spectrum^6^. However, while these lamps can be used to disinfect unoccupied spaces, direct exposure to conventional germicidal UV lamps in occupied public spaces is not possible since direct exposure to these germicidal lamp wavelengths can be a health hazard, both to the skin and eye^7–10^.

By contrast far-UVC light (207 to 222 nm) has been shown to be as efficient as conventional germicidal UV light in killing microorganisms^11^, but studies to date^12–15^ suggest that these wavelengths do not cause the human health issues associated with direct exposure to conventional germicidal UV light. In short (see below) the reason is that far-UVC light has a range in biological materials of less than a few micrometers, and thus it cannot reach living human cells in the skin or eyes, being absorbed in the skin stratum corneum or the ocular tear layer. But because viruses (and bacteria) are extremely small, far-UVC light can still penetrate and kill them. Thus far-UVC light potentially has about the same highly effective germicidal properties of UV light, but without the associated human health risks^12–15^. Several groups have thus proposed that far-UVC light (207 or 222 nm), which can be generated using inexpensive excimer lamps, is a potential safe and efficient anti-microbial technology^12–18^ which can be deployed in occupied public locations.

The biophysically-based mechanistic basis to this far-UVC approach^12^ is that light in this wavelength range has a very limited penetration depth. Specifically, far-UVC light (207–222 nm) is very strongly absorbed by proteins through the peptide bond, and other biomolecules^19,20^, so its ability to penetrate biological materials is very limited compared with, for example, 254 nm (or higher) conventional germicidal UV light^21,22^. This limited penetration is still much larger than the size of viruses and bacteria, so far-UVC light is as efficient in killing these pathogens as conventional germicidal UV light^12–14^. However, unlike germicidal UV light, far-UVC light cannot penetrate either the human stratum corneum (the outer dead-cell skin layer), nor the ocular tear layer, nor even the cytoplasm of individual human cells. Thus, far-UVC light cannot reach or damage living cells in the human skin or the human eye, in contrast to the conventional germicidal UV light which can reach these sensitive cells^7–10^.

In summary far-UVC light is anticipated to have about the same anti-microbial properties as conventional germicidal UV light, but without producing the corresponding health effects. Should this be the case, far-UVC light has the potential to be used in occupied public settings to prevent the airborne person-to-person transmission of pathogens such as coronaviruses.

We have previously shown that a very small dose (2 mJ/cm^2^) of far-UVC light at 222 nm was highly efficient in inactivating aerosolized H1N1 influenza virus^23^. In this work we explore the efficacy of 222 nm light against two airborne human coronaviruses: alpha HCoV-229E and beta HCoV-OC43. Both were isolated over 50 years ago and are endemic to the human population, causing 15–30% of respiratory tract infections each year^24^. Like SARS-CoV-2, the HCoV-OC43 virus is from the beta genus^25^.

Here we measured the efficiency with which far-UVC light inactivates these two human coronaviruses when exposed in aerosol droplets of sizes similar to those generated during sneezing and coughing^26^. As all coronaviruses have comparable physical and genomic size, a critical determinant of radiation response^27^, we hypothesized that both viruses would respond similarly to far-UVC light, and indeed that all coronaviruses will respond similarly.

## Results

### Inactivation of human coronaviruses after exposure to 222 nm light in aerosols infectivity assay

**Figure 1:**
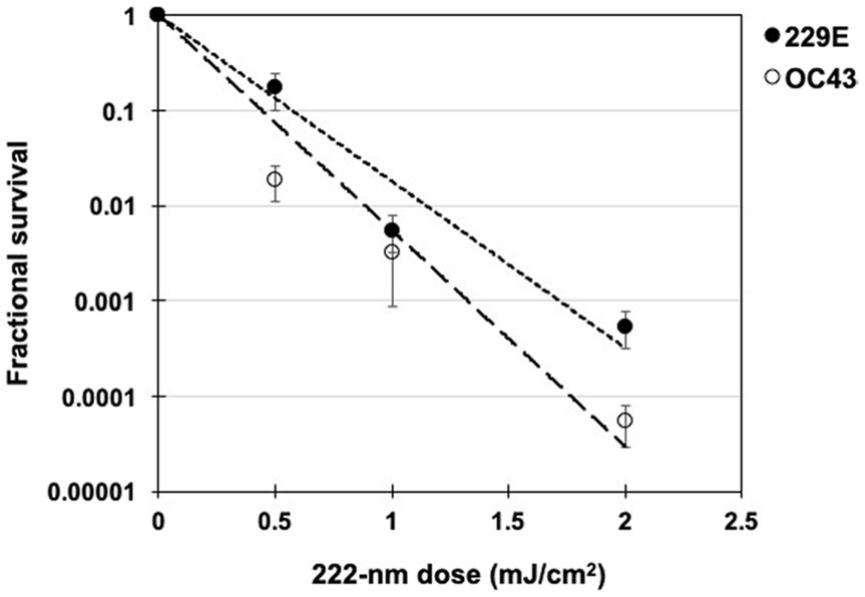
Coronavirus survival as function of the dose of far-UVC light. Fractional survival, PFU_UV_ / PFU_controls,_ is plotted as a function of the 222-nm far-UVC dose. The results are reported as the estimate plaque forming units (PFU)/ml using the conversion PFU/ml = 0.7 TCID_50_^29^ by applying the Poisson distribution. Values are reported as mean ± SEM from multiple experiments (n = 3 alpha HCoV-229E and n = 4 for beta HCoV-OC43); the lines represent the best-fit regressions to equation (1) (see text and Table 1).

We used a standard approach to measure viral inactivation, assaying coronavirus infectivity in human host cells (normal lung cells), in this case after exposure in aerosols to different doses of far-UVC light. We quantified virus infectivity with the 50% tissue culture infectious dose TCID_50_ assay^28^, and estimated the corresponding plaque forming units (PFU)/ml using the conversion PFU/ml = 0.7 TCID_50_^29^. Figure 1 shows the fractional survival of aerosolized coronaviruses HCoV-229E and HCoV-OC43 expressed as PFU_UV_/PFU_controls_ as a function of the incident 222-nm dose. Robust linear regression (Table 1) using iterated reweighted least squares^30^ indicated that the survival of both genera alpha and beta is consistent with a classical exponential UV disinfection model (R^2^ = 0.86 for HCoV-229E and R^2^ = 0.78 for HCoV-OC43). For the alpha coronavirus HCoV-229E, the inactivation rate constant (susceptibility rate) was *k* = 4.1 cm^2^/mJ (95% confidence intervals (C.I.) 2.5–4.8) which corresponds to an inactivation cross-section (or the dose required to kill 90% of the exposed viruses) of D_90_ = 0.56 mJ/cm^2^. Similarly, the susceptibility rate for the beta coronavirus HCoV-OC43 was *k* = 5.9 cm^2^/mJ (95% C.I. 3.8–7.1) which corresponds to an inactivation cross section of D_90_ = 0.39 mJ/cm^2^.

**Table 1:** Linear regression parameters for normalized ln[S] [survival] values (equation 1) as the dependent variable and UV dose (D, mJ/cm^2^) as the independent variable. k is the UV inactivation rate constant or susceptibility factor (cm^2^/mJ). The linear regression was performed with the intercept term set to zero representing the definition of 100% relative survival at zero UV dose. The coronavirus inactivation cross section, D_90_ (the UV dose that inactivates 90% of the exposed virus) was calculated using D_90_ = − ln[1 − 0.90]/k.

Human coronavirus type	*k* (cm^2^/mJ)	*k* 95% confidence interval		p value	R^2^	D_90_ (mJ/cm^2^)
		*Lower*	*Upper*			
**HCoV-229E**	4.1	2.5	4.8	0.0003	0.86	**0.56**
**HCoV-OC43**	5.9	3.8	7.1	0.0001	0.78	**0.39**

### Viral integration assay

**Figure 2:**
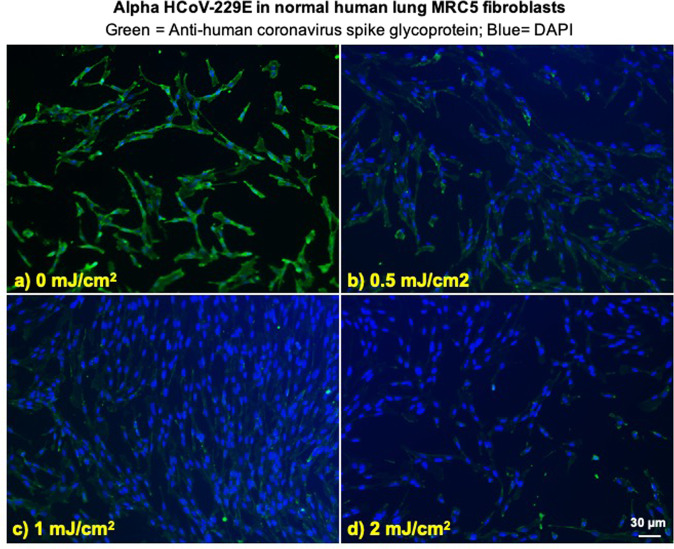
Infection of human lung cells from irradiated aerosolized alpha HCoV-229E as function of dose of far-UVC light. Representative fluorescent images of MRC-5 normal human lung fibroblasts infected with human alphacoronavirus 229E exposed in aerosolized form. The viral solution was collected from the BioSampler after running through the aerosol chamber while being exposed to (**a**) 0, (**b**) 0.5, (**c**) 1 or (**d**) 2 mJ/cm^2^ of 222-nm light. Green fluorescence qualitatively indicates infected cells (Green = Alexa Fluor-488 used as secondary antibody against anti-human coronavirus spike glycoprotein antibody; Blue = nuclear stain DAPI). Images were acquired with a 10× objective; the scale bar applies to all the panels in the figure.

**Figure 3:**
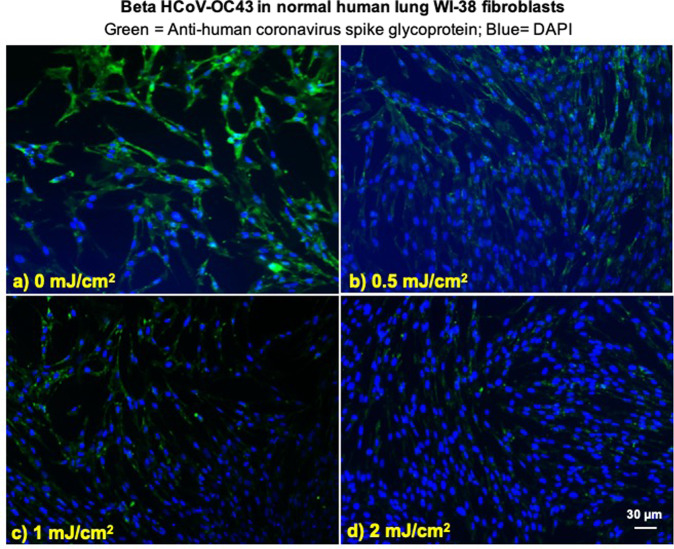
Infection of human lung cells from irradiated aerosolized beta HCoV-OC43 as function of dose of far-UVC light. Representative fluorescent images of WI-38 normal human lung fibroblasts infected with human betacoronavirus OC43 exposed in aerosolized form. The viral solution was collected from the BioSampler after running through the aerosol chamber while being exposed to (**a**) 0, (**b**) 0.5, (**c**) 1 or (**d**) 2 mJ/cm^2^ of 222-nm light. Green fluorescence qualitatively indicates infected cells (Green = Alexa Fluor-488 used as secondary antibody against anti-human coronavirus spike glycoprotein antibody; Blue = nuclear stain DAPI). Images were acquired with a 10× objective; the scale bar applies to all the panels in the figure.

We investigated integration of the coronavirus in human lung host cells, again after exposure in aerosols to different doses of far-UVC light. Figures 2 and 3 show representative fluorescent 10x images of human lung cells MRC-5 and WI-38 incubated, respectively, with HCoV-229E (Fig. 2) and HCoV-OC43 (Fig. 3), which had been exposed in aerosolized form to different far-UVC doses. The viral solution was collected from the BioSampler after running through the aerosol chamber while being exposed to 0, 0.5, 1 or 2 mJ/cm^2^ of 222-nm light. Cells were incubated with the exposed virus for one hour, the medium was replaced with fresh infection medium, and immunofluorescence was performed 24 hours later. We assessed the human cell lines for expression of the viral spike glycoprotein, whose functional subunit S2 is highly conserved among coronaviruses^31,32^. In Figs. 2 and 3, green fluorescence (Alexa Fluor-488 used as secondary antibody against anti-human coronavirus spike glycoprotein antibody) qualitatively indicates infection of cells with live virus, while the nuclei were counterstained with DAPI appearing as blue fluorescence. For both alpha HCoV-229E and beta HCoV-OC43, exposure to 222-nm light reduced the expression of the viral spike glycoprotein as indicated by a reduction in green fluorescence.

## Discussion

The severity of the 2020 COVID-19 pandemic warrants the rapid development and deployment of effective countermeasures to reduce indoor person-to-person transmission. We have developed a promising approach using single-wavelength far-UVC light at 222 nm generated by filtered excimer lamps, which inactivates airborne viruses without inducing biological damage in exposed human cells and tissue^11–18^. The approach is based on the biophysically-based principle that far-UVC light, because of its very limited penetration in biological materials, can traverse and kill viruses and bacteria which are typically micrometer dimensions or smaller, but it cannot penetrate even the outer dead-cell layers of human skin, nor the outer tear layer on the surface of the human eye^12^.

In this work we have used an aerosol irradiation chamber to test the efficacy of 222-nm far-UVC light to inactivate two aerosolized human coronaviruses, beta HCoV-OC43 and alpha HCoV-229E. As shown in Fig. 1, inactivation of the two human coronavirus by 222-nm light follows a typical exponential disinfection model, with an inactivation constant for HCoV-229E of *k* = 4.1 cm^2^/mJ (95% C.I. 2.5–4.8), and *k* = 5.9 cm^2^/mJ (95% C.I. 3.8–7.1) for HCoV-OC43. These values imply that 222 nm UV light doses of only 1.7 mJ/cm^2^ or 1.2 mJ/cm^2^ respectively produce 99.9% inactivation  (3-log reduction) of aerosolized alpha HCoV-229E or beta HCoV-OC43. A summary of *k* values and the corresponding D_90_, D_99_, and D_99.9_ values we have obtained for coronaviruses is shown in Table 2, together with our earlier results for aerosolized H1N1 influenza virus^23^. The relatively small difference in influenza A (H1N1) and human coronaviruses sensitivity to 222-nm light is likely attributable to differences in structure, genome size, and nucleic acid configuration^33^. It is also important to note that the previous results with H1N1 virus utilized a fluorescent focus assay to assess virus survival^23^ in contrast to this work which used the TCID_50_ assay. While both assays are widely used to accurately determine viral infectivity^34^, the former employs immunofluorescence to detect a specific viral antigen, instead of depending on cytopathic effects as in the TCID_50_ assay. Because the assays differ in methods and principles, some variance is expected between these two techniques.

**Table 2:** Estimated *k*, D_99_, and D_99.9_ values for exposure to 222 nm far-UVC light for alphacoronavirus HCoV-229E, betacoronavirus HCoV-OC43, and influenza A (H1N1).

Species	*k* (cm^2^/mJ)	D_90_ (mJ/cm^2^)	D_99_ (mJ/cm^2^)	D_99.9_ (mJ/cm^2^)
HCoV-229E	4.1	0.56	1.1	1.7
HCoV-OC43	5.9	0.39	0.78	1.2
Influenza A (H1N1)*^†^	1.8	1.3	2.6	3.8

^*^D_99_, and D_99.9_ values for influenza A (H1N1) denote extrapolated values, as these doses were not used during testing^23^.

^†^Our previous work with H1N1 utilized the fluorescent focus assay^23^, while the current work with coronaviruses used the TCID_50_ assay.

The results suggest that both of the studied coronavirus strains have similar high sensitivity to far-UVC inactivation. Robust linear regression produced overlapping 95% confidence intervals for the inactivation rate constant, *k*, of 2.5 to 4.8 cm^2^/mJ and 3.8 to 7.1 cm^2^/mJ respectively for the 229E and OC43 strains. As all human coronaviruses have similar genomic sizes which is a primary determinant of UV sensitivity^27^, it is reasonable to expect that far-UVC light will show similar inactivation efficiency against all human coronaviruses, including SARS-CoV-2. The data obtained here are consistent with this hypothesis.

It is useful to compare the performance of far-UVC light with conventional germicidal (peak 254 nm) UVC exposure. We are aware of only one such study^35^, which used an aerosolized murine beta coronavirus. The study reported a D_88_ of 0.599 mJ/cm^2^, which others^4^ have used to estimate the D_90_ for the virus with 254 nm light as 0.6 mJ/cm^2^. This value is similar to those estimated in the current work (see Table 2), suggesting similar inactivation efficiency of 222 nm far-UVC and conventional germicidal 254 nm UVC for aerosolized coronavirus, and providing further support for the suggestion that all coronaviruses have similar sensitivities to UV light.

The sensitivity of the coronaviruses to far-UVC light, together with extensive safety data even at much higher far-UVC exposures^12–18^, suggests that it may be feasible and safe to have the lamps providing continuous low-dose far-UVC exposure in public places – potentially reducing the probability of person-to-person transmission of coronavirus as well as other seasonal viruses such as influenza. In fact there is a regulatory limit as to the amount of 222 nm light to which the public can be exposed, which is 23 mJ/cm^2^ per 8-hour exposure^36,37^. Based on our results here for the beta HCoV-OC43 coronavirus, continuous far-UVC exposure at this regulatory limit would result in 90% viral inactivation in approximately 8 minutes, 95% viral inactivation in approximately 11 minutes, 99% inactivation in approximately 16 minutes and 99.9% inactivation in approximately 25 minutes. Thus continuous airborne disinfection with far-UVC light at the currently regulatory limit would provide a major reduction in the ambient level of airborne virus in occupied indoor environments.

In conclusion, we have shown that very low doses of far-UVC light efficiently kill airborne human coronaviruses carried by aerosols. A dose as low as 1.2 to 1.7 mJ/cm^2^ of 222-nm light inactivates 99.9% of the airborne human coronavirus tested from both genera beta and alpha, respectively. As all human coronaviruses have similar genomic size, a key determinant of radiation sensitivity^27^, it is likely that far-UVC light will show comparable inactivation efficiency against other human coronaviruses, including SARS-CoV-2.

Together with previous safety studies^12–18^ and our earlier studies with aerosolized influenza A (H1N1)^23^, these results suggest the utility of continuous low-dose-rate far-UVC light in occupied indoor public locations such as hospitals, transportation vehicles, restaurants, airports and schools, potentially representing a safe and inexpensive tool to reduce the spread of airborne-mediated viruses. While staying within the current regulatory dose limits, low-dose-rate far-UVC exposure can potentially safely provide a major reduction in the ambient level of airborne coronaviruses including SARS-CoV-2.

## Methods

### Viral strains

HCoV-229E (VR-740) and HCoV-OC43 (VR-1558) were propagated in human diploid lung MRC-5 fibroblasts (CCL-171) and WI-38 (CCL-75), respectively (all from ATCC, Manassas, VA). Both human cell lines were grown in MEM supplemented with 10% Fetal Bovine Serum (FBS), 2 mM L-alanyl-L-glutamine, 100 U/ml penicillin and 100 μg/ml streptomycin (Sigma-Aldrich Corp. St. Louis, MO, USA). The virus infection medium consisted of MEM or RPMI-1640 plus 2% heat inactivated FBS for HCoV-229E and HCoV-OC43, respectively. The viral strains were propagated by inoculation of flasks containing 24-hours old host cells, which were 80–90% confluent. After one hour incubation, the cell monolayer was washed and incubated in fresh infection medium for three or four days at 35 °C for HCoV-229E and at 33 °C for HCoV-OC43. The supernatant containing the working viral stock was then collected by centrifugation (300 *g* for 15 minutes). The virus titer was determined by 50% tissue culture infective dose TCID_50_ by assessing cytopathic effects (CPE), which were scored at a bright field microscope (10×) as vacuolization of cytoplasm, cell rounding and sloughing.

### Benchtop aerosol irradiation chamber

A one-pass, dynamic aerosol/virus irradiation chamber was used to generate, expose, and collect aerosol samples as previously described^23^. Viral aerosols were generated by adding a virus solution in a high-output extended aerosol respiratory therapy nebulizer (Westmed, Tucson, AZ) and operating using an air pump with an input flow rate of 11 L/min. Virus flowed into the chamber and was mixed with dry and humidified air to maintain humidity between approximately 50–70%. The relative humidity, temperature, and aerosol particle size distribution were monitored throughout operation. Aerosol was exposed to far-UVC light and finally collected using a BioSampler (SKC Inc., Eighty Four, PA).

The far-UVC lamp was positioned approximately 22 cm away from the UV exposure chamber and directed at the 26 cm × 25.6 cm × 254 μm UV-transmitting plastic window (TOPAS 8007 × 10, TOPAS Advanced Polymers Inc., Florence, KY). Consistent with our previous experiments using this chamber^23^, the flow rate through the system was 12.5 L/min. The volume of the UV exposure region was 4.2 L so each aerosol was exposed for approximately 20 seconds as it traversed the window. The entire irradiation chamber was contained in a biosafety level 2 cabinet and all air inputs and outputs were equipped with HEPA filters (GE Healthcare Bio-Sciences, Pittsburgh, PA) to prevent unwanted contamination from entering or exiting the system.

### Irradiation chamber performance

The custom irradiation chamber simulated the transmission of aerosolized viruses produced via human coughing and breathing. The chamber operated at an average relative humidity of 66% and an average temperature of 24 °C across all runs. The average particle size distribution was 83% between 0.3 μm and 0.5 μm, 12% between 0.5 μm and 0.7 μm, and 5% >0.7 μm (Table 3). Aerosolized viruses were efficiently transmitted through the system as evidenced from the control (zero exposure) showing clear virus integration (Figs. 2 and 3, top left panels).

**Table 3:** Example of particle size distributions from humans during various activities are given^26^ along with the averaged measured values for this work.

		Particle Size Distribution	
		<1.0 μm	>1.0 μm
**Papineni*et al.***^26^	Coughing	83–91%	9–16%
	Mouth Breathing	83–95%	4–16%
	Nose Breathing	83–100%	0–16%
	Talking	77–88%	11–22%
**This work**	**0.3–0.5 μm**	**0.5–0.7 μm**	**>0.7 μm**
	83%	12%	5%

### Far-UVC lamp and dosimetry

The far-UVC source used in this study was a 12 W 222-nm KrCl excimer lamp module made by USHIO America (Item #9101711, Cypress, CA). The lamp is equipped with a proprietary optical filtering window to reduce lamp emissions outside of the 222 nm KrCl emission peak. The lamp was positioned 22 cm away from the exposure chamber window and directed at the center of the window. Optical power measurements were performed using an 818-UV/DB low-power UV enhanced silicon photodetector with an 843-R optical power meter (Newport, Irvine, CA). Dosimetry was performed prior to starting an experiment to measure the fluence within the chamber at the position of the aerosol.

The distance between the lamp and the irradiation chamber permitted a single lamp to uniformly irradiate the entire exposure window area. Measurements using the silicon photodetector indicated an exposure intensity of approximately 90 μW/cm^2^ across the exposure area. The chamber is equipped with a reflective aluminum surface opposite of the exposure window. As in our previous work with this chamber^23^, the reflectivity of this surface was approximately 15%. We have therefore conservatively estimated the intensity across the entire exposure area to be 100 μW/cm^2^. With the lamp positioned 22 cm from the window and given the 20 seconds required for an aerosol particle to traverse the exposure window, we calculated the total exposure dose to a particle to be 2 mJ/cm^2^. We used additional sheets of UV transmitting plastic windows to uniformly reduce the intensity across the exposure region to create different exposure conditions. While in our previous work with these sheets we measured a transmission closer to 65%^23^, for these tests we measured the 222-nm transmission of each sheet to be approximately 50%. This decrease in transmission is likely due to the photodegradation of the plastic over time^4^. The addition of one or two sheets of the plastic covering the exposure window decreases the exposure dose to 1 and 0.5 mJ/cm^2^, respectively.

### Experimental protocol

As previously described^23^, the virus solution in the nebulizer consisted of 1 ml of Modified Eagle’s Medium (MEM, Life Technologies, Grand Island, NY) containing 10^7^–10^8^ TCID_50_ of coronavirus, 20 ml of deionized water, and 0.05 ml of Hank’s Balanced Salt Solution with calcium and magnesium (HBSS^++^). The irradiation chamber was operated with aerosolized virus particles flowing through the chamber and the bypass channel for 5 minutes prior to each sampling, in order to establish the desired RH value. Sample collection was initiated by changing air flow from the bypass channel to the BioSampler using the set of three way valves. The BioSampler was initially filled with 20 ml of HBSS^++^ to capture the aerosol. During each sampling time, which lasted for 30 minutes, the inside of the irradiation chamber was exposed to 222-nm far-UVC light entering through the plastic window. Variation of the far-UVC dose delivered to aerosol particles was achieved by inserting additional UV-transparent plastic films as described above thereby delivering the three test doses of 0.5, 1.0 and 2.0 mJ/cm^2^. Zero-dose control studies were conducted with the excimer lamp turned off. After the sampling period was completed the solution from the BioSampler was used for the virus infectivity assays.

### Virus infectivity assays

#### TCID_50_

We used the 50% tissue culture infectious dose assay to determine virus infectivity^28^. Briefly, 10^5^ host cells were plated in each well of 96-well plates the day prior the experiment. Cells were washed twice in HBSS^++^ and serial 1:10 dilutions in infection medium of the exposed virus from the BioSampler was overlaid on cells for two hours. The cells were then washed twice in HBSS^++^, covered with fresh infection medium, and incubated for three or four days at 34 °C. Cytopathic effects (CPE) were scored at a bright field microscope (10×) as vacuolization of cytoplasm, cell rounding and sloughing. The TCID_50_ was calculated with the Reed and Muench method^28,38^. To confirm the CPE scores, the samples were fixed in 100% methanol for five minutes and stained with 0.1% crystal violet. The results are reported as the estimate of plaque forming units (PFU)/ml using the conversion PFU/ml = 0.7 TCID_50_ by applying the Poisson distribution^29^.

#### *Immunofluorescence*

To assess whether increasing doses of 222-nm light reduced the number of infected cells, we performed a standard fluorescent immunostaining protocol to detect a viral antigen in the host human cells^23^. Briefly, 2 × 10^5^ host cells (MRC-5 cells for HCoV-229E and WI-38 for HCoV-OC43) were plated in each well of 48-well plates the day prior the experiment. After running through the irradiation chamber for 30 minutes, 150 μl of virus suspension collected from the BioSampler was overlaid on the monolayer of host cells. The cells were incubated with the virus for one hour, then washed three times with HBSS^++^, and then incubated overnight in fresh infection medium. Infected cells were then fixed in 100% ice cold methanol at 4 °C for 5 minutes and labeled with anti-human coronavirus spike glycoprotein (40021-MM07, Sino Biologicals US Inc., Chesterbrook, PA) 1:200 in HBSS^++^ containing 1% bovine serum albumin (BSA; Sigma-Aldrich Corp. St. Louis, MO, USA) at room temperature for one hour with gentle shaking. Cells were washed three times in HBSS^++^ and labeled with goat anti-mouse Alexa Fluor-488 (Life Technologies, Grand Island, NY) 1:800 in HBSS^++^ containing 1% BSA, at room temperature for 30 minutes in the dark with gentle shaking. Following three washes in HBSS^++^, the cells were stained with Vectashield containing DAPI (4′,6-diamidino-2-phenylindole) (Vector Laboratories, Burlingame, CA) and observed with the 10× objective of an Olympus IX70 fluorescent microscope equipped with a Photometrics PVCAM high-resolution, high-efficiency digital camera and Image-Pro Plus 6.0 software (Media Cybernetics, Bethesda, MD). For each 222-nm dose and virus genus, the representative results were repeated twice. For each sample, up to ten fields of view of merged DAPI and Alexa Fluor-488 images were acquired.

### Data analysis

The surviving fraction (*S*) of the virus was calculated by dividing the fraction PFU/ml at each UV dose (PFU_UV_) by the fraction at zero dose (PFU_controls_): *S* = PFU_UV_/PFU_controls_. Survival values were calculated for each repeat experiment and natural log (ln) transformed to bring the error distribution closer to normal^39^. Robust linear regression using iterated re-weighted least squares (IWLS)^40,41^ was performed in *R* 3.6.2 software using these normalized ln[*S*] values as the dependent variable and UV dose (D, mJ/cm^2^) as the independent variable. Using this approach, the virus survival [*S*] was described by first-order kinetics according to the equation^4^:

1 $$\mathrm{ln}[{\rm{S}}]=-k\times D$$

where *k* is the UV inactivation rate constant or susceptibility factor (cm^2^/mJ). The regression was performed with the intercept term set to zero representing the definition of 100% relative survival at zero UV dose, separately for each studied virus strain. The data at zero dose, which by definition represent ln[S] = 0, were not included in the regression. Uncertainties (95% confidence intervals, CI) for the *k* parameter for each virus strain were estimated by bootstrapping for each regression method because bootstrapping may result in more realistic uncertainty estimates, compared with the standard analytic approximation based on asymptotic normality, in small data sets such as those used here (n = 3 HCoV-229E and n = 4 for HCoV-OC43). Goodness of fit was assessed by coefficient of determination (R^2^). Analysis of residuals for autocorrelation and for heteroskedasticity was performed using the Durbin-Watson test^42^ and Breusch-Pagan test (implemented by *lmtest R* package)^43^, respectively. Parameter estimates (*k*) for each virus strain were compared with each other based on the 95% CIs and directly by *t*-test, using the sample sizes, *k* values, and their standard errors. The virus inactivation cross section, D_90_, which is the UV dose that inactivates 90% of the exposed virus, was calculated as D_90_ = − ln[1 − 0.90]/*k*. Other D values were calculated similarly.

## Data Availability

The dataset file generated during and/or analysed during the current study is available in the Open Science Framework (OSF) repository, identifier: DOI 10.17605/OSF.IO/KGZAF.
