# Author Correction: Far-UVC light (222 nm) efficiently and safely inactivates airborne human coronaviruses

**DOI:** 10.1038/s41598-021-97508-9

**Published:** 2021-09-27

**Authors:** Manuela Buonanno, David Welch, Igor Shuryak, David J. Brenner

**Affiliations:** Center for Radiological Research, Columbia University Irving Medical Center, New York, NY 10032 USA

Correction to: *Scientific Reports* https://doi.org/10.1038/s41598-020-67211-2, published online 24 June 2020

The Authors wish to clarify the Competing Interests. The text,

“The authors declare the following pending patent: Patent Title: “Apparatus, method and system for selectively affecting and/or killing a virus”. Applicant: The Trustees of Columbia University in the City of New York. Inventors: Gerhard Randers-Pehrson, David Jonathan Brenner, Alan Bigelow. Application #: US20180169279A1. Aspect of manuscript covered in patent application: Use of filtered 222 nm UV light to kill viruses URL: https://patents.google.com/patent/US20180169279A1/en?oq=20200085984.”

now reads as follows:

“D.J.B has a granted patent entitled ‘Apparatus, method and system for selectively affecting and/or killing a virus’ (US10780189B2), that relates to the use of filtered 222 nm UV light to inactivate viruses. In addition, D.J.B has an ongoing non-financial collaboration with Eden Park Illumination, and the authors’ institution, Columbia University, has licensed aspects of UV light technology to USHIO Inc.”

The original Article has been corrected.

